# Woman

**Published:** 1893-06

**Authors:** Thomas Campbell


### WOMAN.

---

> "The world was sad, the garden was a wild,
> And man, the hermit, sighed—till woman smiled."
>
> THOMAS CAMPBELL.

Woman! Sweet woman! What magic in the name!

Who can think of her without reverence, or speak of her without emotion. What myriads of beauteous visions and happy memories arise before us when we contemplate this most wonderful of all God's creatures. Her very being is enshrouded in unfathomable mystery.

Perhaps it was upon a summer's day, somewhere in Paradise, in some shaded dell, beside some rippling streamlet that passed merrily by. The sweet perfume of rarest flowers was borne hither and thither upon the gentle zephrous winds. Birds of rich and varied plumage were flitting here and there, and anon would dip their gaudy wings in the crystal fount, and then perched on pendant bough and swinging branch would fill the air with note of heavenly melody. Likely enough did father

Adam succumb to a hypnotism so divine as this. Then, perchance, for a moment, the birds were silent, and the streamlet passed noiselessly by, as if in reverent awe at the great event, and in the holy hush of the sacred hour there was achieved the grandest triumph known to surgery. No genius has ever conceived its design, and no intellect has ever mastered its technique. It was done by the almighty hand of God, and no eye but his witnessed its performance. Any other save the Divine Presence would have been a profanation and a sacrilege. In this notable aperation, it is said that Adam lost a rib, but gained a companion, counsellor and friend, and woman was given to the world. God had saved his best work for his last, and made her the culmination of his creative power and the expression of his most beautiful thought.

Adam awoke and there stood before him, in nature's beauteous adornment and bewitching attitude, a figure that no painter's brush or poet's pen can e'er portray. I fancy he stood transfixed, motionless and amazed ere he could determine whether he beheld an apparition or a reality, or that he had ever known what beauty was before. "Her every motion was full of grace, and every gesture full of love." "When I approach her loveliness, so absolute she seems, and in herself complete, so well to know her own, that what she wills to do or say seems wisest, virtuoüsest, discreetest, best."

As no eye witnesseed her formation, there is no tongue to tell the secret of her power. We only know she is a problem that defies solution. Who can fathom the mystery of her being, or comprehend the limits of her influence. Her power is subtle and imperceptible, yet pervades and dominates humanity everywhere. She is at once passive, yet in the highest degree active, indefinable yet definite, ephemeral yet constant, weak yet strong, always gentle yet never a negative quality. Wonderful paradox this, that so sets at naught all power of analysis. But however that may be, she yet remains the greatest gift of God to man. Her influence comes to him like the dew that gently falls at the silent hour of midnight, refreshing his strength, buoying up his drooping spirits and preparing him anew for duty and high endeavor. Her power is the hand of succor, and like the warm summer sunshine rescuing nature from winter's icy grasp and bringing it back to life and beauty, so does she from gloom, darkness and despair, bring man back to sunshine; hope and joy.

From the Adamic days down to the present, the world has ever felt her impress, and history itself is but a record of her majestic sway. Man in all ages, and in every clime has been a devout worshipper at her shrine, and paid homage to her imperial personage. " For her, strong men delight to weave garlands of love, and on her head place coronets of devotion. In the deeds of heroism that light up the pages of history, woman's figure stands out paramount to all others. The tale of suffering is but the story of her devotion. She is the physician's strongest ally, as well as his best patient.

Her self-sacrifice in time of war and disaster, and her work upon the battlefilds, carrying comfort to the suffering and cheer to the dying, too truly attest the genuineness of her patriotism. This has been demonstrated a thousand times, but never so nobly, or so gloriously at it was by the women of the South when our fair land was well nigh drenched in fratricidal blood. Then it was that woman rose to the zenith of her glory, and there was written the most brilliant and enduring chapter in in the history of her devotion. All honor, all glory to the fair women of the South ! Incomparable in heroism ; Unexcelled in virtue ; Undying in devotion !

I have spoken of her power and influence, her patriotism, her her heroic devotion. But how can I leave her thus, when this happy spring time, the green foliage of the trees, the sweet warble of the mocking birds, the blossoming of the flowers, that send a thousand delicate odors out upon the air, all conspire to fill me with an inspiration that makes my "fancy lightly turn to thoughts of love."

Who can resist her sweet enchantment or tell whence cometh this strange quickening of the heart beats. Who can dispel the sensation that stirs the soul when her gentle voice breaks forth in strains of heavenly melody. There is no light that glows like the radiance of her smile, and no twinkling star has e'er outshone the sparkle of her eyes, and in their liquid depths me thinks I sometimes see visions of a fairer world than this. There is no hue or tint of rose or pink can outrival the blush upon her cheek. Her words are like "apples of gold in pictures of silver." Her heart is but the prism that reflects the beauties of a heavenly light, and reveals the attributes of God to man. Happy is he who is honored by her confidence, and blessed by her tender love.

Friend, lover, comrade, sweetheart—who dares ignore the power of her charms, or deny allegiance to her throne?

"My kingdom is my sweetheart's face,

And these the boundaries I trace.

Northward her forehead fair,

Beyond a wilderness of hair,

A rosy cheek to East and West

Her little mouth

The Sunny South.

It is the South that I love best.

Her eyes two crystal lakes

Rippling with light

Caught from the sun by day,

The stars by night.

The dimples in

Her cheeks and chin

Are snares which love hath set.

And I have fallen in."

Of all the joys that bless mankind, the sweetest is the joy of a new found love.

"The one thing in this world that is constant, the one peak that rises above all clouds, the one window in which light forever burns, the one star that darkness cannot quench is woman's love. This is the real love that subdues the earth, the love that wrought all miracles in art, that gives us music all the way from the cradle song to the grand symphony that bears the soul away on wings of fire. A love that is greater than power, sweeter than life, stronger than death.

Typical of its refreshing and life-giving power, emblematical of its purity and its strength is this glass of crystal water which I drink in honor to the name of fair and lovely woman. May she live, and ever by her hallowed influence bless, comfort, uplift and ennoble mankind. On her head may Heaven send its sweetest benedictions.

A response to toast delivered at Banquet of Georgia Medical Association at Americus, Ga., on the evening of April 20th, 1893, by Dr. R. E. L. Baruum, Savannah, Ga.

### BOOKS AND PAMPHLETS RECEIVED.

Physical Diagnosis, by A. L. Loomis, M. D. Published by William Wood & Co., New York.

Surgical Dressings, Aseptic and Antiseptic, by S. W. Williams, M. D. (Reprint.)

A Remarkable Respiration, Record in Infantile Pneumonia, Acute Enlargement of the Thyroid Gland, Angio-Neurotic Oedema, by W. A. Edwards, M. D., San Diego, Cal. (Reprint.)

Preparation of the City of Brooklyn for the Anticipated Epidemic of Cholera in 1884 and 1885, by Joseph H. Raymond, M. D., Ex-Commissioner of Health, Brooklyn, N. Y. (Reprint.)

Mustard in Therapeutics, by A. H. Laidlaw, M. D., and Frederick Laidlaw, M. D., New York. (Reprint.)

Descriptive Sketch and Drawings of two Cases of Symmetrically placed Opacities of the Corneae, occurring in Mother and Son, by Charles A. Oliver, M. D., Philadelphia, Pa. (Reprint.)

The Climatic Treatment of Phthisis, with a description of the climate of Asheville, N. C., by Karl Von Ruck, M. D., Asheville, N. C. (Reprint.)

On the Relation of Eczema to Disturbances of the Nervous System, by L. Duncan Bulkley, A. M., M. D., attending physician to the New York Skin and Cancer Hospital.

Clinical Study and Analysis of 1,000 Cases of Psoriasis, by L. Duncan Bulkley, A. M., M. D., New York.

How to Operate for Hemorrhoids, by Charles B. Kelsey, M. D., New York.

